# Cryptogenic Organizing Pneumonia: A Unique Case and Literature Review

**DOI:** 10.7759/cureus.25793

**Published:** 2022-06-09

**Authors:** Boyd Davis, Tahir Allauddin

**Affiliations:** 1 Pulmonary and Critical Care, Southside Medical Center, Petersburg, USA

**Keywords:** case report, diagnostic work-up, treatment, bronchiolitis obliterans organizing pneumonia, cryptogenic organizing pneumonia

## Abstract

Pneumonia is one of the most common pathologies seen in the inpatient setting. The rapid response to treat febrile patients with infiltrates on chest x-ray has reduced hospital length of stay and hospital costs. However, the automatic reaction to treat all infiltrates and opacities seen on a chest x-ray as pneumonia can be costly. This report presents the case of a patient suspected initially of having pneumonia, who was unresponsive to broad-spectrum antibiotics. A 58-year-old woman presented with dyspnea on exertion and a nonproductive cough. Her chest x-ray showed dense right-sided coalescent opacities encompassing the entirety of the right lung. Flexible bronchoscopy biopsy specimens revealed the cause to be cryptogenic organizing pneumonia. This case highlights the diverse set of pulmonary pathologies that can mimic pneumonia and should be suspected in cases of antibiotic-resistant suspected pneumonia patients.

## Introduction

Cryptogenic organizing pneumonia (COP), known formerly as bronchiolitis obliterans organizing pneumonia, is a form of idiopathic diffuse interstitial lung disease. This rare form of pneumonia has no known direct cause but is associated with many caustic inhaled agents and pathogens. This report presents the case of a patient initially suspected of having pneumonia who was unresponsive to broad-spectrum antibiotics and ultimately diagnosed with COP.

## Case presentation

A 58-year-old woman with a history of chronic obstructive pulmonary disease presented to the emergency department with primary concerns of dyspnea on exertion and constant nonproductive cough, progressively worsening over the last two weeks. Associated symptoms included subjective fever, chills, decreased appetite, and dizziness. She had a body temperature of 100°F (37.8°C), heart rate of 108 beats per minute (bpm), respiratory rate of 28 breaths per minute, oxygen saturation of 87% on room air, and peripheral blood pressure measurement of 135/92 mmHg. Her complete blood cell count revealed leukocytosis at 18,000/μL. Her laboratory results showed she was hyperchloremic (112 mEq/L) with low bicarbonate levels (15 mEq/L; Table 1). Subsequent arterial blood gas analysis revealed metabolic acidosis with respiratory compensation (Table 2). On lung auscultation, we noted crackles over the entirety of the right lung fields and basilar crackles on the left. Pro-brain natriuretic peptide levels were within the reference range (Table 1). Her chest x-ray revealed an unusual case of dense right-sided coalescent opacities encompassing the entirety of the right lung, sparing the left lung. Her rapid test result for coronavirus disease 2019 (COVID-19) was negative, and the patient denied any recent COVID-19 infection. The patient was admitted to the telemetry floor under the premise of sepsis and was started on a broad-spectrum antibiotic (piperacillin/tazobactam) and 2 L of oxygen delivered via nasal cannula.

**Table 1:** Patient's laboratory results µL: microliter; g: gram; dL: deciliter; mEq: milliequivalent; L: liter; pg: picogram

Analyte	Patient Value	Reference Range
Complete Blood Count:		
Red blood cell count	4.2 million cells/µL	3.92–5.13 million cells/µL
Hemoglobin	12.4 g/dL	11.6–15 g/dL
Hematocrit	39.8%	35.5–44.9%
White blood cell count	17,000 cells/µL	3,400–9,600 cells/µL
Platelet count	180,000 cells/µL	157,000–371,000 cells/µL
Basic Metabolic Panel:		
Sodium	138 mEq/L	135–145 mEq/L
Potassium	3.9 mEq/L	3.7–5.2 mEq/L
Chloride	112 mEq/L	96–106 mEq/L
Bicarbonate	15 mEq/L	22–29 mEq/L
Creatinine	0.8 mEq/dL	0.6–1.3 mg/dL
Blood urea nitrogen	11 mEq/dL	6–20 mEq/dL
Brain natriuretic peptide	70 pg/mL	< 100 pg/mL

**Table 2:** Patient's arterial blood gas results mmHg: millimeters of mercury; mEq: milliequivalents; L: liter

Analyte	Patient Value	Reference Range
pH	7.3	7.35–7.45
Partial pressure of oxygen	70 mmHg	80–100 mmHg
Partial pressure of carbon dioxide	30 mmHg	35–45 mmHg
Bicarbonate	17 mEq/L	22–29 mEq/L

Upon admission, high-resolution computed tomography (CT) indicated dense patchy consolidations in the entirety of the right lung with sparse involvement of the left lung peripherally. The CT also indicated a small pleural effusion in the right pleural space. By day three of admission, the patient showed minimal signs of clinical improvement on broad-spectrum antibiotics. Her sputum cultures and stains were negative, and blood cultures were not growing any bacteria after 48 hours, leading to the decision to perform a flexible bronchoscopy with bronchoalveolar lavage (BAL) for further diagnostic workup. BAL revealed mixed cellularity with neutrophils, lymphocytes, and eosinophils. Lymphocytes were the predominant cell, encompassing over 40% of counted cells. A lung biopsy was performed in the right superior lobe at a point where the bronchiole was partially obstructed by an unknown growth. A histopathological examination of this sample demonstrated granulation tissue buds extending into the bronchiole tissue, mild mononuclear cell interstitial inflammation, and foamy macrophages in the lung immediately surrounding the granulation tissue. These findings are consistent with COP; however, a surgical lung biopsy would be required for a definitive diagnosis. After a lengthy discussion with the treatment team and the patient, the patient decided to undergo treatment for COP rather than proceed with the surgical biopsy, given her radiographic evidence and clinical status. She was started on high-dose prednisone at 60 mg once daily and 500 mg of azithromycin daily. Four days after starting this treatment plan, the patient experienced overall improvement. Her oxygen saturation was 95%, her respiratory rate was 18 breaths per minute, her heart rate was 90 bpm, and she was functionally able to ambulate the hospital halls without experiencing more than mild dyspnea.

## Discussion

COP is a rare disease; a major teaching hospital in Canada measured the cumulative prevalence at 6.7 cases per 100,000 hospital admissions [1]. COP is an acute inflammatory and proliferative process distinguished by its reversible intraalveolar fibrous proliferation. This acute process can often be reversed with immunosuppressive and antiinflammatory agents. The reversible nature separates COP from other fibrotic interstitial lung pathologies [2,3].

Chest x-rays and high-resolution CT scans are first-line diagnostic workups for COP, often showing patchy diffuse consolidations mostly involving bilateral lower zones and bilateral patchy peripherally located consolidations or ground-glass opacities [4]. The diagnostic workup typically continues with bronchoscopy and BAL. Our patient had characteristic features of COP on BAL and bronchial biopsy. To make a definitive diagnosis, a surgical lung biopsy must be performed. Wedge biopsies are preferably obtained from at least two lobes with radiographic evidence of COP involvement. However, the risk of these surgical procedures must be weighed against the strength of the patient's clinical picture. If the clinical and radiographic evidence is convincing of COP, treatment can begin without a lung biopsy after a discussion with the patient [4].

There have been no controlled trials comparing medications or duration of treatment in COP. Treatment regimens are based on current consensus guidelines [5], centered around glucocorticoids and macrolide antibiotics. Glucocorticoids decrease the transcription of proinflammatory mediators by binding to their nuclear receptors. This acute decrease in inflammatory mediators makes glucocorticoids effective in treating COP, an acute inflammatory reaction to an external pulmonary irritant. Glucocorticoid therapy normally leads to clinical improvement in the first 24 to 72 hours, with radiologic evidence of COP normally disappearing within three months [6,7]. The initial dose is given for two to four weeks; over the next six to 12 months, the dose should steadily be tapered to zero to avoid adrenal crisis [6]. In adjunct with glucocorticoids, macrolide antibiotic therapy is an option supported by case reports and small, retrospective series that have suggested macrolide antibiotics have antiinflammatory properties useful in the treatment of COP [8,9]. A long-term retrospective study demonstrated that macrolide antibiotic use in COP reduces the incidence of disease relapse [9].

## Conclusions

This case report highlights the clinical and radiographic features of COP, an uncommon lung pathology. It offers an additional differential diagnosis for patients with suspected pneumonia unresponsive to antimicrobial therapy. This case report also highlights the limited research focused on the treatment of COP. The current treatment of COP is based on consensus guidelines. This case report illustrates the useful nature of macrolide antibiotics in COP. A controlled clinical trial based on macrolide therapy in COP, especially compared to glucocorticoid therapy alone, could yield crucial clinical knowledge in the treatment of COP.
